# Development of an Underwater Adaptive Penetration System for In Situ Monitoring of Marine Engineering Geology

**DOI:** 10.3390/s24175563

**Published:** 2024-08-28

**Authors:** Miaojun Sun, Zhigang Shan, Wei Wang, Shaopeng Zhang, Heyu Yu, Guangwei Cheng, Xiaolei Liu

**Affiliations:** 1 Zhejiang Engineering Research Center of Marine Geotechnical Investigation Technology and Equipment, Zhejiang Huadong Geotechnical Investigation & Design Institute Co., Ltd., Powerchina Huadong Engineering Co., Ltd., Hangzhou 311122, China; sun_mj2@hdec.com (M.S.); shan_zg@hdec.com (Z.S.); wang_w20@hdec.com (W.W.); 2 Shandong Provincial Key Laboratory of Marine Environment and Geological Engineering, Ocean University of China, Qingdao 266100, China; millerzhangsp@163.com (S.Z.); zghydxhyyu@163.com (H.Y.); guangwei09@163.com (G.C.)

**Keywords:** offshore wind power, seabed liquefaction, seabed scouring, in situ monitoring, multi-sensor, penetration system

## Abstract

In recent years, offshore wind farms have frequently encountered engineering geological disasters such as seabed liquefaction and scouring. Consequently, in situ monitoring has become essential for the safe siting, construction, and operation of these installations. Current technologies are hampered by limitations in single-parameter monitoring and insufficient probe-penetration depth, hindering comprehensive multi-parameter dynamic monitoring of seabed sediments. To address these challenges, we propose a foldable multi-sensor probe and establish an underwater adaptive continuous penetration system capable of concurrently measuring seabed elevation changes and sediment pore water pressure profiles. The reliability of the equipment design is confirmed through static analysis of the frame structure and sealed cabin. Furthermore, laboratory tests validate the stability and accuracy of the electrical and mechanical sensor measurements. Preliminary tests conducted in a harbor environment demonstrate the system’s effectiveness.

## 1. Introduction

As an important marine resource and clean energy, offshore wind energy is considered an effective solution to achieve greenhouse gas emission reduction [1,2,3]. By the end of 2022, the total installed capacity of offshore wind power in the world was 64.3 GW, of which China accounted for 48.9% [4]. However, at the same time, the frequency and scale of offshore engineering geological disasters caused by extreme weather are increasing, posing a serious threat to the location, construction, operation, and maintenance of offshore wind farms [5,6,7].

Offshore disasters caused by extreme weather manifest in two ways. Firstly, forces such as wind, waves, and currents directly impact engineering structures. Under combined external loads, wind turbines are susceptible to damage [8,9]. Secondly, environmental loads from waves and currents affect seabed sediments. This can lead to scouring around piles and sediment liquefaction, causing wind turbines to destabilize and topple [10,11]. Zhang et al. [12], for example, found in a field investigation of an offshore wind farm in the East China Sea that the scouring depth of monopile was 7.74 m (1.3D). Zhang et al. [13] carried out dynamic simulations, and the results showed that dynamic loads in the marine environment would cause pore water pressure to concentrate around the pile and destroy the sediments.

The performance loss or facility damage caused by the long-term operation of wind turbines in the marine environment can be easily detected and repaired in time, but engineering geological disasters such as scouring or liquefaction at the pile foundation need to be monitored by more methods.

For typical engineering geological disasters in offshore wind farms, such as scouring and liquefaction, in situ monitoring technologies based on various principles have been developed in the world. At present, sensors based on acoustic and optical principles are often used to realize the in situ monitoring of seabed scouring. Acoustic rangefinders proposed by Turner et al. [14], sediment erosion sensors based on a dense array of light-sensitive cells developed by Hu et al. [15], and automated optical in situ sensors proposed by Matos et al. [16] can overcome the limitations of long-term data acquisition, but the monitoring effectiveness of these instruments is environmentally relevant and susceptible to external factors such as solid suspended particles and hydrodynamic conditions. For the change of pore water pressure in sediments, many wind power piles are installed with pore water pressure sensors in the sediments, which can realize long-term monitoring of pore pressures around pile foundations during the piling process as well as in the operation of wind turbines [17,18,19]. However, only pore water pressure data around the pile can be obtained, and once the sensor is damaged, it will be difficult to replace, hindering long-term in situ monitoring tasks. Penetrating in situ monitoring equipment, represented by pore water pressure probes, has also been used to monitor the change process of seabed pore water pressure [20,21,22]. Urgeles et al. [23] placed a Pop Up Pore Pressure Instrument developed by Universitat de Barcelona in Barcelona, Spain inside the sediment to study the relationship between volcanic activity and changes in pore water pressure. Lucking et al. [24] used probe free-fall penetration to obtain the dynamic distribution of pore water pressure in the shallow sediments. Xu et al. [25] conducted long-term monitoring of the change of pore water pressure in sediments in the Yellow River Delta of China under wave action through an 8-m-long pore water pressure probe.

In summary, current in situ monitoring of sediment typically employs probe penetration. To obtain real-time data from deeper sediments, a longer probe is needed. At present, the penetration equipment equipped with the long-stroke probe is generally high and heavy, which makes the equipment difficult to transport and operate underwater. Internationally renowned seabed probe penetration equipment, such as the DW-ROSON100 developed by Van den Berg in Heerenveen, Netherlands, the SEACALF developed by Fugro in Den Haag, Netherlands, and the MANTA-200 developed by Geomil in Moordrecht, Netherlands, can achieve penetration depths of 40 m [26,27], but their weight and height are still a serious problem. The PeneVector, developed by Peneson in Wuhan, China, can penetrate to a depth of 42 m [28], but its weight reaches 3500 kg, and the probe needs to be spliced on water. The seabed probe penetration equipment developed by Zhejiang University can solve the problem of a too-long probe [29], and its penetration depth can reach 10 m, but its weight can reach 5000 kg and its height can reach 3500 mm. And the above-mentioned equipment is only used for the detection of physical and mechanical properties such as cone tip resistance and side friction. Additionally, although there are specialized seabed elevation and sediment pore water pressure instruments mentioned above, obtaining multi-parameter changes such as pore water pressure and seabed elevation requires deploying numerous devices in phases, and it is difficult to obtain long-term monitoring data at the same point on the seabed simultaneously. Therefore, there is an urgent need to develop integrated multi-parameter monitoring equipment to address engineering geological disasters like seabed scouring and sediment liquefaction in offshore wind farms.

Based on this, this study offers the underwater adaptive penetration system for in situ long-term multi-parameter monitoring (UAPS/ILMM), which enables long-stroke penetration of monitoring probe and simultaneous multi-parameter data acquisition. Additionally, the static analysis of the equipment and the laboratory test of the sensor are carried out to verify the system’s feasibility and accuracy. Finally, the system is further tested in a harbor environment, and relevant data are collected.

## 2. System Design and Working Principles

### 2.1. Overview of the System

**Figure 1:**
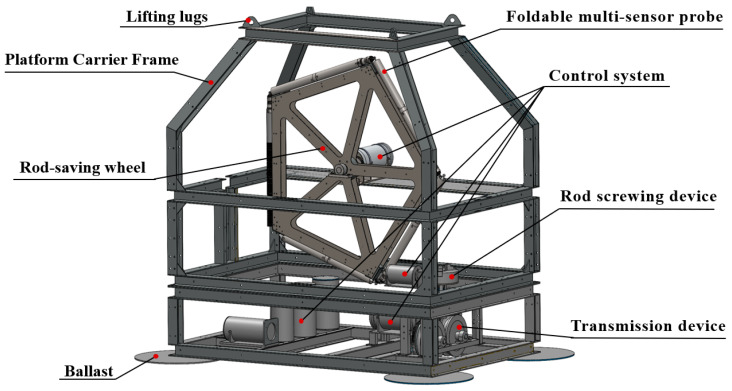
Design schematic of UAPS/ILMM.

UAPS/ILMM is developed to solve the technical problems of single monitoring data and short penetration stroke of the current seabed in situ monitoring equipment. As shown in Figure 1, it adopts a frame structure design as a whole, which is mainly composed of three parts: a platform carrier of the system, a foldable multi-sensor probe, and an underwater adaptive penetration device.

This system has the characteristics of simple structure, large penetration, and small size. The specific technical indexes are shown in Table 1, which can be used for long-term in situ stable monitoring of seabed elevation, pore water pressure, and excess pore water pressure of sediment through the combination of electricity and mechanics.

**Figure 2:**
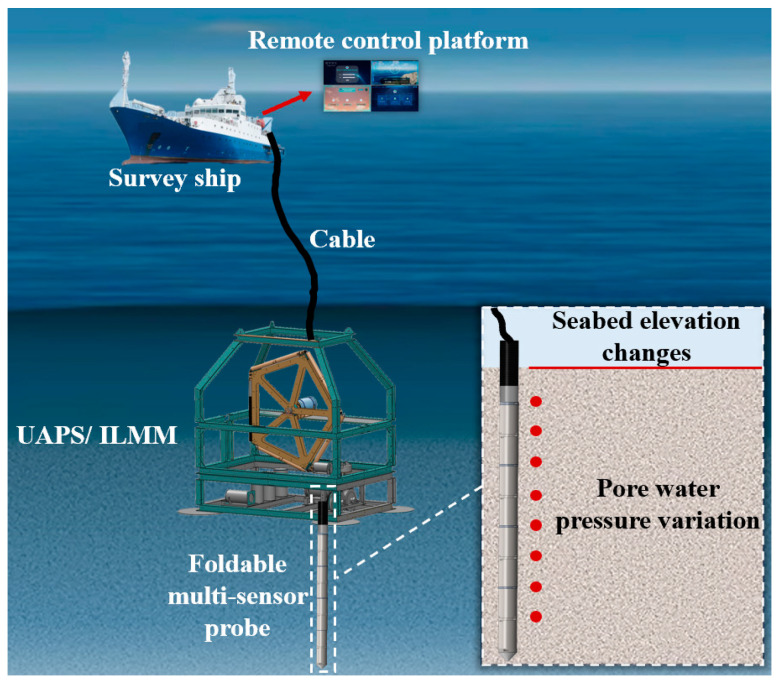
Schematic of UAPS/ILMM in the offshore sea. Note this figure is not to scale, a red arrow indicates that the survey ship is equipped with a remote control platform.

The working principle of UAPS/ILMM is shown in Figure 2. The whole system is transported to the sea area to be measured by the survey ship, and it is deployed to the seabed through the armored photoelectric composite cable. Then, the probe penetration command is issued through the deck control unit, and at the same time, information such as the working state of the equipment and the video image in the penetration process are also transmitted to the deck control unit in real time through the photoelectric composite cable. After the penetration is completed, the probe will carry out long-term in situ stable monitoring of sediment pore water pressure and seabed elevation changes within 5 m below the seabed surface and transmit them to the deck control unit in real time through the photoelectric composite cable.

### 2.2. Platform Carrier of the System

As the basic frame of the whole equipment, the platform carrier is mainly used to provide a support base to carry important components such as penetration devices, the foldable multi-sensor probe, data acquisition, motor nacelles, and cameras, which is the premise for the safe and reliable operation of the whole system underwater.

This study designs a platform carrier with a rectangular frame structure, measuring 2.8 m by 1.8 m in cross-section and standing 2.8 m tall. To minimize the impact of ocean currents on the system’s stability, the upper half of the platform is optimally designed to reduce water flow influence, ensuring minimal drift in strong currents. This design enhances system safety and reduces the risk of probe damage caused by excessive displacement due to turbulent disturbances. The upper layer houses a pentagonal rod-saving wheel with 1 m sides, connected to the frame via a central axle. This wheel can rotate precisely within the upper space, driven by a motor. For structural stability, four horizontal beams are added around the upper layer. The lower layer accommodates key components such as the penetration device, data-acquisition unit, motors, and cameras. Placing most of the system’s components at the lower layer effectively lowers the center of gravity, enhancing stability during seabed operations.

The platform carrier is welded and formed by 316 L stainless steel material, which has good mechanical properties, processing properties, and corrosion resistance, can resist seawater erosion, has low manufacturing cost, and can accept various forms of welding, ensuring the integrity and compactness of the system structure.

### 2.3. Foldable Multi-Sensor Probe

Traditional seabed static cone penetration probes extend by adding rods above water [30,31], which can become unstable if they are too long. To address this, the multi-sensor probe is divided into five 1-m rods. These rods are bent five times into a pentagonal shape and stored on a 316 L steel rod-saving wheel. Each rod segment is precisely threaded for easy underwater assembly.

**Figure 3:**
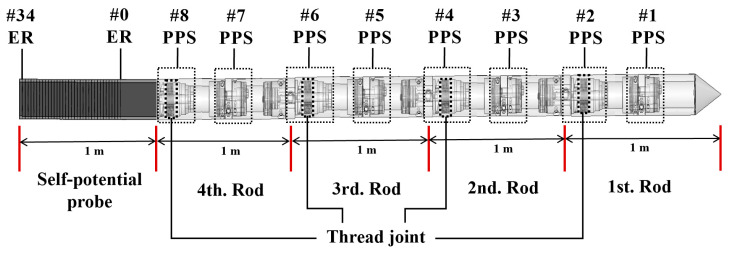
Schematic of foldable multi-sensor probe. Note the red line is used as a segment identifier for the foldable multi-sensor probe, the symbol # in this paper is used to indicate the serial number of PPS or ER.

From a mechanical perspective, the foldable multi-sensor probe consists of a cone tip, eight pore pressure sensors (PPS), and 35 self-potential sensor electrode rings (ER). The fifth rod, which is the self-potential probe, connects to the signal-acquisition and battery compartments on the lower layer of the platform via waterproof connectors, as shown in Figure 3. Integrating pore pressure sensors and self-potential sensors allows for effective measurement of sediment pore water pressure and long-term in situ monitoring of seabed interface characteristics.

The first four rods of the probe are equipped with pore pressure sensors at both the tail and midpoints, using differential fiber Bragg grating sensing technology [22]. This design adapts to the corrosive and low-temperature environment of long-term seabed monitoring. Two water guide pipes are arranged inside the PPS, which are used for transmitting the pore water pressure from the sediment and the hydrostatic pressure from the seabed, respectively. By employing an open differential structure, the difference between the two pressure values mentioned above, that is, the excess pore water pressure, is converted into the spring deformation by using the internal spring tube. As a result, the light waves in the fiber Bragg grating outside of the spring tube are offset, and the corresponding excess pore water pressure can be obtained according to the offset. This system balances sediment pore water pressure with hydrostatic pressure, obtaining high-precision excess pore water pressure data by measuring the difference between hydrostatic pressure from the seabed surface and actual sediment pore water pressure. This configuration allows for the acquisition of eight sets of pore water pressure distributions at 0.5-m intervals within the sediment profile.

**Figure 4:**
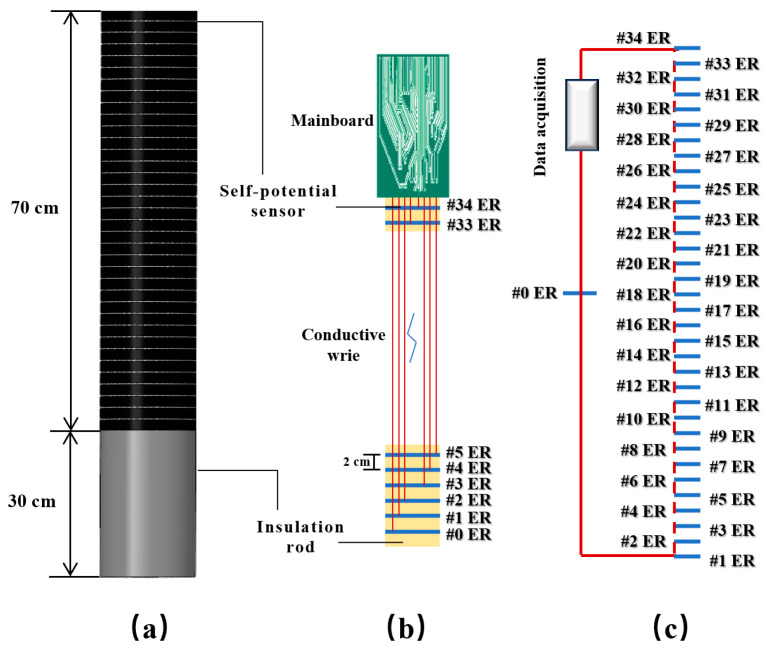
Schematic of self-potential probe. (**a**) Size of self-potential probe, (**b**) The connective wire of the self-potential sensor inside the probe, (**c**) The probe rod measurement method.

As mentioned above, acoustic and optical sensors are susceptible to the water environment above the seabed, so UAPS/ILMM uses ER to monitor the dynamic changes in seabed elevation. Due to the difference in the composition of seawater and sediment, there is a significant difference in the redox environment between the two, and there is a potential difference caused by the self-potential anomaly at the seabed. By forming a battery with a self-potential sensor and a reference sensor, the self-potential value can be measured at different positions. As shown in Figure 4, the fifth rod of the probe, the final segment, is the self-potential probe. This insulated rod is made from polyoxymethylene resin and fitted with 35 self-potential sensors made from 2-mm-thick TC4 titanium alloy, arranged from top to bottom. The #0 ER serves as the reference sensor. Seals between the sensors and the insulated rod maintain an internal environment isolated from external water. Considering the sensor influence and monitoring accuracy between self-potential sensors, the sensor spacing is set at 2 cm, providing a vertical resolution of 2 cm. To avoid signal interference between the self-potential sensors and the pore pressure sensors, a 30-cm empty space is left at the bottom of the self-potential probe.

### 2.4. Underwater Adaptive Penetration Device

**Figure 5:**
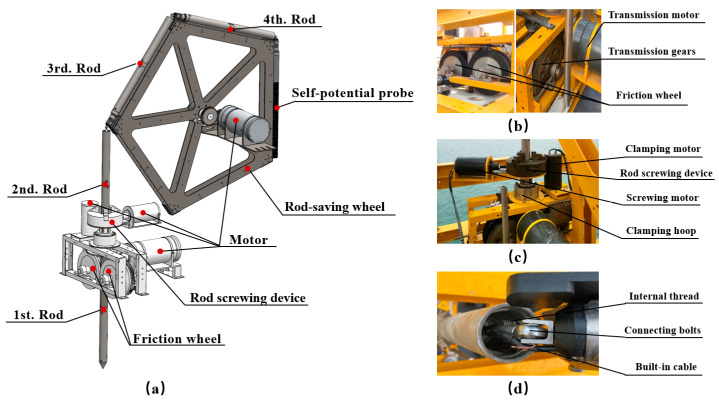
Schematic of the underwater adaptive penetration device. (**a**) Schematic of the penetration device, (**b**) Friction wheel transmission, (**c**) Rod screwing device, (**d**) Connection structure of the rods.

As shown in Figure 5, the underwater adaptive penetration device addresses the issue of excessive overall height due to the length of the probe. By reducing the system’s height and extending the penetration depth, the system ensures overall stability during underwater operations. The system consists of a multi-sensor probe, a pentagonal rod-saving wheel, a rod screwing device, and a friction wheel transmission. The multi-sensor probe is folded into five equal-length segments and stored in the outer groove of the rod-saving wheel. The transmission is designed to uniformly penetrate the probe into the seabed.

Two common methods are hydraulic-driven penetration and friction wheel sliding penetration. The hydraulic method requires additional heavy components like hydraulic cylinders and clamps, which increase the system’s weight and risk of sinking. Therefore, we chose the lighter friction wheel method. As shown in Figure 5b, the friction wheel transmission structure is mainly two friction wheels and a transmission motor. The outer diameter of the friction wheel is 350 mm, the outer ring of the friction wheel has an inward groove, and two friction wheels of the same specification are located in the same vertical plane, which is used to enclose the probe rod and form a channel for the probe to penetrate. There is also a coaxial transmission gear outside the friction wheel, and the penetration force of the motor is transmitted to the two friction wheels through the gear structure to rotate in opposite directions at the same time. Eventually, the friction created between the wheel and the rod drives the probe to penetrate vertically.

Since the probe designed in this paper is foldable, and the probe is divided into five rods of the same length before penetration, a rod screwing device is specially designed in this paper for the rod splicing operation. As shown in Figure 5c, it is mainly composed of a screwing device, a screwing motor, a clamping device, and a clamping motor, wherein the screwing device and the clamping device are, respectively, used for realizing the rotation and clamping operation of two rods.

In particular, in order to facilitate efficient splicing of rods, we did not completely divide the five rods as separate individuals. As shown in Figure 5d, the bolt structure is used to connect the five rods, the two rod-connection sections are, respectively, the structure of internal thread and external thread, and the two rods can be easily connected by using a clamping device and a screwing device. It is worth noting that the bolt structure and the cable are located in the middle of the rod, but they are not connected with the rod by the rigid structure. There is space between them, and the rods can rotate relative to each other and slide radially within a small range. Such a structure ensures that the bolt structure and the cable remain relatively stationary when the rods are spliced.

During the penetration operation, the rod-saving wheel motor is activated to rotate the wheel clockwise, transferring the first rod segment into a set of the friction wheel below via the rod screwing device. The transmission motor drives the gear structure to rotate the friction wheel inward to penetrate the first rod segment into the seabed, while the wheel continues to rotate. When the head of the second rod segment enters the screwing device, the wheel stops rotating. The clamping device clamps the first rod segment, and the screwing device rotates the second rod clockwise to connect the external thread of the second segment with the internal thread of the first segment, forming a single continuous rod. The wheel and transmission motor are then restarted to continue the penetration process. This procedure is repeated until all five rod segments are penetrated. Notably, the fifth self-potential probe does not need to be fully penetrated, as the exposed self-potential sensors can monitor seabed elevation in situ.

## 3. Static Analysis

### 3.1. Static Analysis of the System Structure

To ensure that the platform maintains its mechanical performance and avoids structural deformation under its own weight during underwater operations, a static analysis of the platform’s frame structure will be conducted.

**Figure 6:**
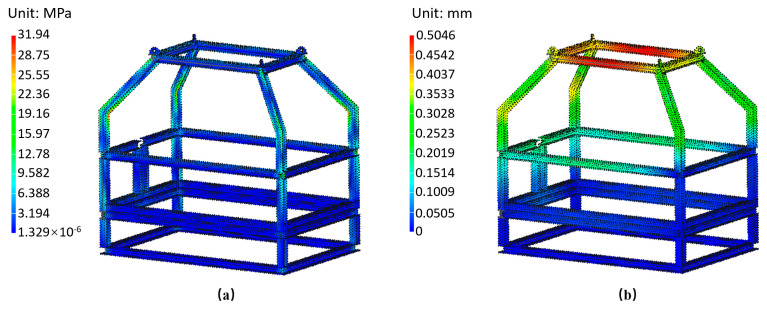
Static analysis of the system structure. (**a**) Stress distribution, (**b**) Total deformation.

The frame structure is designed using stainless steel (316 L), and each weld joint is modeled as bonded to prevent relative sliding or separation. Fixed support constraints are applied to the bottom of the frame, and a vertical load of 2 tons is applied to the four lifting lugs on the top. The analysis calculates the average equivalent stress and total deformation of the frame under its own weight. The results of the static analysis of the platform frame are shown in Figure 6.

The simulation analysis of the platform frame reveals a uniform stress distribution with no significant variations. The maximum stress occurs at the bending point of the upper frame and is 31.94 MPa, which is well below the minimum yield strength of 316 L stainless steel (177 MPa) [32]. Additionally, the maximum deformation of the frame is 0.5046 mm, within acceptable limits. These results indicate that the designed system platform frame has favorable stress conditions and will maintain its mechanical performance effectively.

### 3.2. Static Analysis of the Sealed Cabin

The pressure-sealed cabin, used for controlling underwater equipment, data acquisition, and power supply, is designed to protect internal components from deep-sea pressure and seawater corrosion. To ensure the cabin’s stability and pressure resistance, we selected a cylindrical structure with an outer diameter of 220 mm, an inner diameter of 190 mm, and a height of 500 mm, made from 316 L stainless steel. This design offers uniform stress distribution, ease of installation, and high internal space utilization.

**Figure 7:**
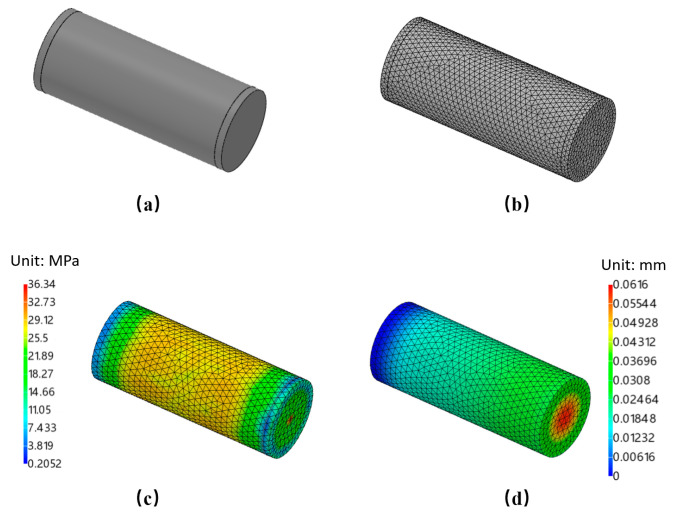
Static analysis of the sealed cabin. (**a**) Simplified model, (**b**) Meshing, (**c**) Stress distribution, (**d**) Total deformation.

To ensure that the pressure-sealed cabin meets the structural strength requirements for underwater operation at depths of 0–500 m, a static analysis of the chamber’s strength was conducted. The analysis involved simplifying the structure, including the watertight connectors. The simplified model and mesh divisions are shown in Figure 7a,b.

The static analysis of the sealed cabin shows that, as shown in Figure 7c,d, under the pressure conditions of 0–500 m depth, the maximum stress is 36.34 MPa, with stress concentration occurring only at the center of the cabin cover, which is within the material’s yield strength. The maximum deformation is 0.062 mm, indicating minimal overall deformation and confirming that the cabin meets the operational requirements for the control and data-acquisition systems.

## 4. Multi-Sensor Reliability Verification

In addition to the safety and stability analysis of the equipment frame structure and the sealing cabin, it is necessary to verify the reliability of data acquisition for the multi-sensor, which is an important part of UAPS/ILMM. The reliability verification of the multi-sensor is mainly divided into the self-potential sensor functionality test and the pore water pressure sensor calibration.

### 4.1. Self-Potential Sensor Functionality Test

**Figure 8:**
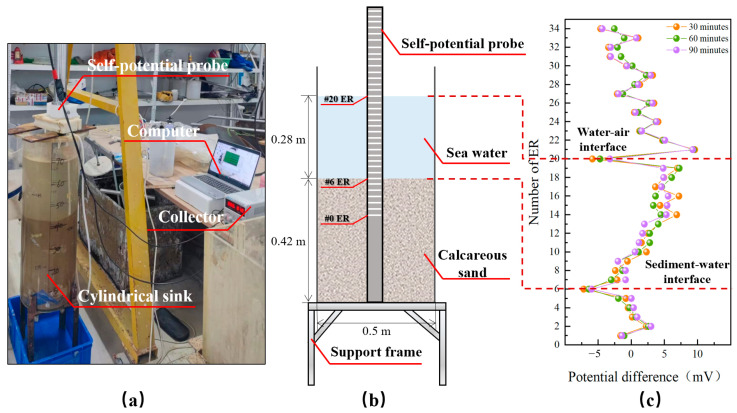
Self-potential sensor functionality test. (**a**) Test facility, (**b**) Diagram of the test process, (**c**) Potential difference test results.

The functional testing of the self-potential sensors aims to determine the differences in self-potential values for various media within a laboratory setting. This test is conducted in a cylindrical glass sink with a height of 0.8 m and a diameter of 0.5 m, shown in Figure 8a. Modulated seawater and sediment (Calcareous sand) are added sequentially. After the sediment is settled naturally to a thickness of 42 cm, #6 ER is positioned at the sediment-water interface. The upper seawater layer, with a thickness of 28 cm, is positioned #20 ER at the water-air interface, as detailed in Figure 8b. After stabilizing the sink environment, three different moments, 30 min later, 60 min later, and 90 min later, are processed and plotted for the difference in measurements between adjacent self-potential sensors, as shown in Figure 8c.

Figure 8c shows that even though the self-potential sensors are in the same medium, there are some deviations in their measurements. However, the differences between sensors generally range from −10 mV to 10 mV, and the magnitude of this difference is more stable in the same medium than at the sediment-water-air interface. Notably, two distinct abrupt changes in self-potential can be observed after the sediment settled and stabilized: the upper change occurred between #20 ER and #21 ER, corresponding to a sink height of 70 cm to 72 cm, and the lower change occurred between #6 ER and #7 ER, corresponding to a height of 40 cm to 42 cm. These findings align well with the actual conditions. The analysis demonstrates that the developed self-potential probe effectively identifies the water-air and sediment-water interfaces with good measurement stability.

### 4.2. Pore Water Pressure Sensor Calibration

**Figure 9:**
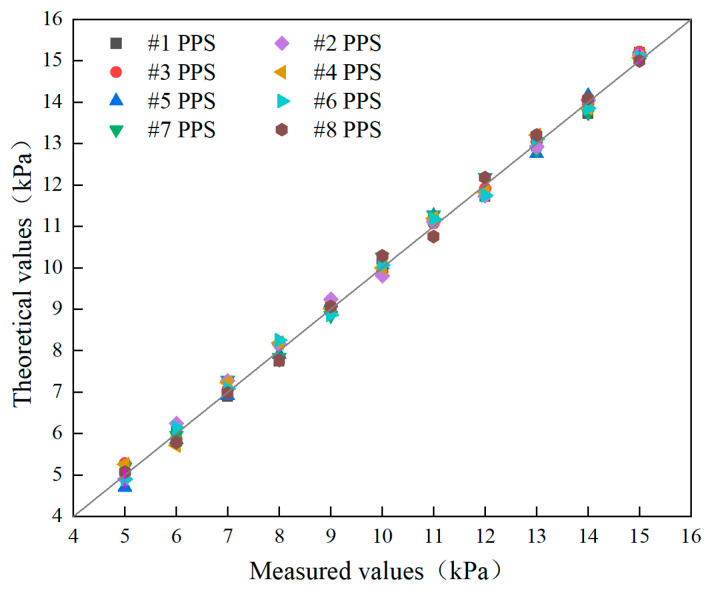
Pore water pressure sensor test results.

Calibration of the pore water pressure sensors can be conducted using a variable head experiment in an indoor sink to determine the correlation between the measured values and theoretical values. Saturated pore water pressure sensors are placed in a controlled seawater environment with varying water heads, from a depth of 50 cm, across 10 levels with 10 cm increments each. Compared with theoretical values, the result is shown in Figure 9.

Figure 9 clearly shows that while the measurements from the eight pore water pressure sensors on the foldable multi-sensor probe exhibit some deviation from theoretical values, the deviation ranges between −0.29 kPa and 0.31 kPa. The correlation between the measured values and theoretical values from #1 PPS to #8 PPS is around 0.998, indicating that the developed sensors provide accurate pore water pressure measurements, meeting the requirements for in situ monitoring of seabed sediments.

## 5. Harbor Test

**Figure 10:**
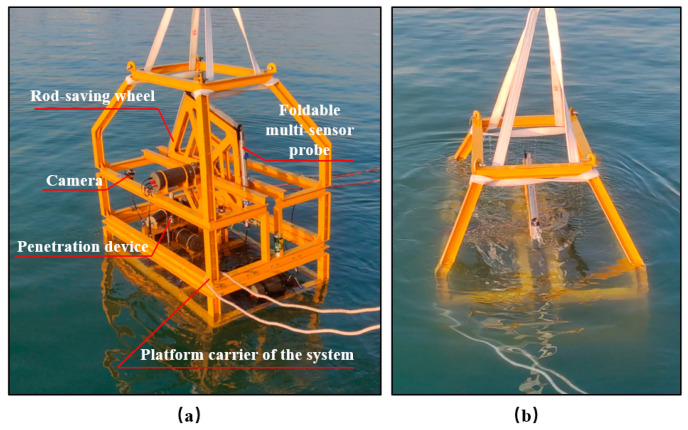
Harbor testing of UAPS/ILMM. (**a**) Deployment, (**b**) Recovery.

From 7 December to 9 December 2022, the UAPS/ILMM was tested for seabed engineering geological environment monitoring in the harbor at the National Deep Sea Center in Qingdao, China. The seabed sediments in this area are primarily silt, characterized by a light gray color and a fine, uniform texture. The water depth in the harbor is consistently around 9–11 m, with depth contours parallel to the shoreline. Physical photos of the UAPS/ILMM system during testing are shown in Figure 10. 

During this period, the system’s underwater segmented penetration, recovery, and data-acquisition functions were comprehensively evaluated, and effective in situ measurements of self-potential and pore pressure were obtained, demonstrating the system’s feasibility and reliability.

Prior to the harbor test, we conducted essential evaluations of the UAPS/ILMM, including pressure-strength tests, sealing tests, and circuit tests. To mitigate the impact of air on pressure transmission, pore water pressure sensors in the probe were fully saturated indoors. After completing the indoor assembly and debugging of the system, the entire setup was transported to the harbor site.

**Figure 11:**
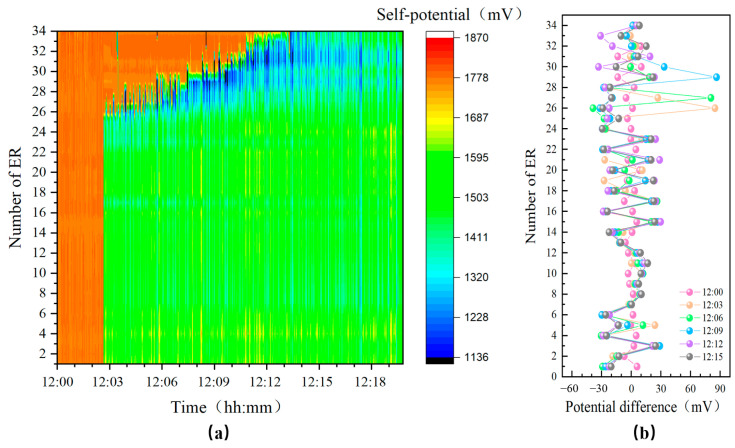
Real-time data measured by self-potential probe during UAPS/ILMM deployment. (**a**) Self-potential profile, (**b**) Potential difference.

Figure 11 illustrates the change in self-potential as the probe transitions from air to seawater. Since the probe is stored in a rod-saving wheel and does not enter the seawater environment vertically, #0 ER to #25 ER were submerged almost simultaneously. Per the evaluations, #26 ER to #34 ER clearly recorded the dynamic changes at the water-air interface. Around 12:02 on 8 December, #0 ER to #25 ER were submerged, and #26 ER began to contact seawater, causing an immediate drop in self-potential. This trend continued to reflect on the subsequent sensors until all sensors were submerged by 12:12.

**Figure 12:**
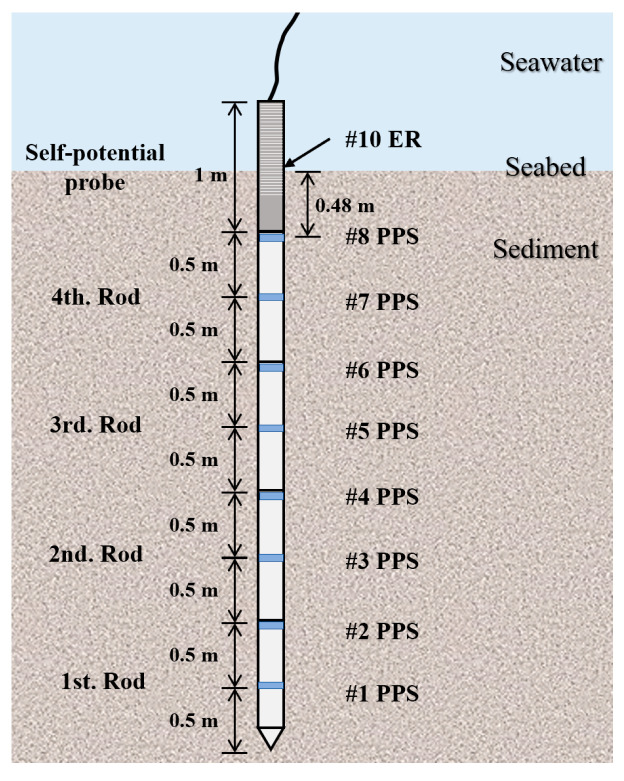
Diagram of the foldable multi-sensor probe penetration.

According to the penetration settings of the foldable multi-sensor probe, to effectively monitor seabed elevation changes, #10 ER and the sensors above it were fully exposed to the seawater environment. Since the probe only penetrated up to #0 to #9 ER, the actual penetration depth of the probe was 4.48 m. Thus, the depth of the #8 pore water pressure sensor (#8 PPS) from the seabed surface was approximately 0.48 m, as shown in Figure 12.

**Figure 13:**
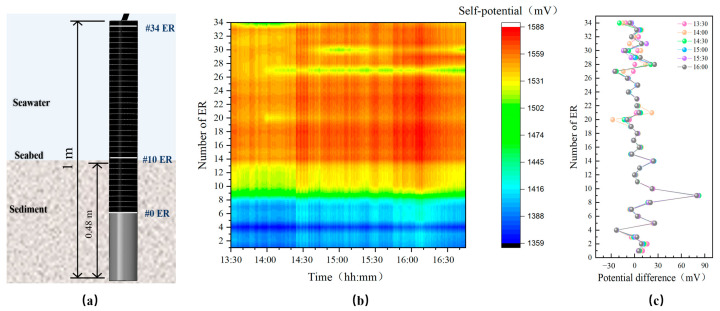
Real-time data measured by self-potential probe after UAPS/ILMM deployment. (**a**) The position of the ER at the sediment-water interface, (**b**) Self-potential profile, (**c**) Potential difference.

Figure 13 shows the actual monitoring data from the probe after penetration. It is evident that there are significant self-potential differences between #8 ER, #9 ER, and #10 ER, consistent with the expected penetration depth settings, where #9 ER is at the seabed surface and #10 ER and above sensors are in seawater. At the seabed interface, the self-potential values fluctuated between 1490 mV and 1505 mV, with no significant variations. This indicates that from 13:30 to 16:30, the seabed did not exhibit erosion or sedimentation exceeding the measurement accuracy range of 2 cm.

**Figure 14:**
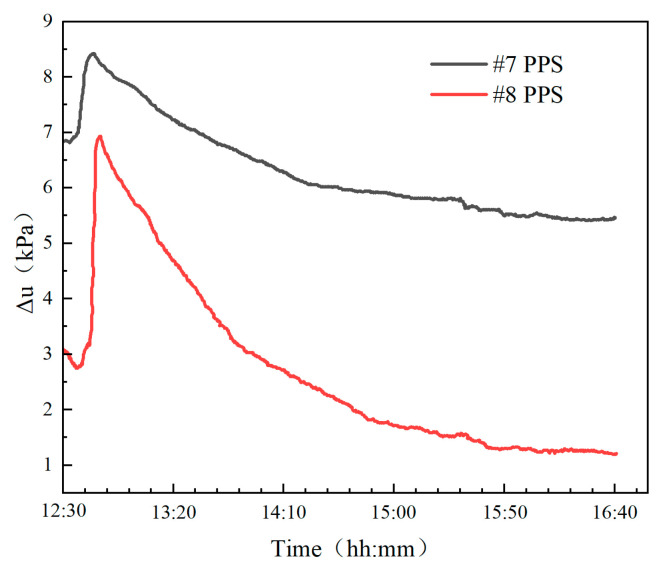
The excess pore water pressure monitoring results.

During the penetration process, the foldable multi-sensor probe experienced malfunctions due to compression from numerous gravel particles in the harbor sediments, resulting in damage to some sensors and loss of data. Figure 14 shows the actual monitoring data from the #7 and #8 pore water pressure sensors (#7 PPS and #8 PPS), which are close to the seabed surface, where the excess pore water pressure is the difference between the hydrostatic pressure transmitted from the seabed surface and the pore water pressure at the depth of the sensor.

As shown in Figure 14, the excess pore water pressure caused by probe penetration peaks after 840 s, with peaks of 8.43 kPa and 6.93 kPa at #7 PPS and #8 PPS, respectively. After that, the excess pore water pressure begins to decay and enters the real-time response stage of the excess pore pressure caused by environmental stress. In particular, because the port sediment is mainly composed of silt, the excess pore water pressure dissipates slowly.

## 6. Related Works Comparison

Table 2 compares the UAPS/ILMM proposed in this paper with other in situ monitoring systems for marine engineering geology. A comprehensive comparison is made in terms of data transmission, power transmission, monitoring objects, structural features, penetration depth, long-term real-time monitoring, and deployment flexibility. Data transmission and power transmission refer to whether the system has cables for real-time transmission. The monitoring objects mainly refer to the changes in the characteristics of seawater, seabed and sediment, such as suspended sediment concentration, seabed elevation, and sediment pore water pressure. The structural features are mainly representative of the weight and size of the system. Long-term real-time monitoring refers to whether the system has the ability to monitor for a long time and have a continuous power supply. Deployment flexibility is primarily the operational convenience of the system for underwater deployment and penetration.

Common seabed monitoring systems can effectively monitor multiple parameters such as suspended sediment concentration and seabed elevation, and are easy to deploy, but their data- and power-transmission methods are self-contained, and it is difficult to obtain sediment characteristics. The sediment pore water pressure monitoring system is capable of obtaining real-time pore water pressure profiles, but its data- and power-transmission methods are also self-contained, and the probe penetration depth is shallow. Although the in situ test system can penetrate into deeper strata and obtain real-time geological conditions at the time of penetration, it is difficult to obtain long-term sediment-monitoring results, the convenience of deployment is medium, and their weight is generally greater than 1450 kg and also higher than 2.8 m. Comparatively speaking, the SEEGeo developed by Ocean University of China in Qingdao, China can obtain long-term real-time monitoring results of sediment characteristics, and it is easy to deploy, but it still has the problem of a shallow penetration depth of 3.5 m and an inability to obtain seabed elevation changes, and it weighs up to 2800 kg and has an overall height of up to 2.2 m. In summary, the UAPS/ILMM proposed in this paper can simultaneously monitor the change characteristics of seabed elevation and sediment pore water pressure, the penetration depth is relatively deep, at least 5 m, and the rods can be added according to the actual situation, so that the overall weight is as light as 1450 kg, the total height is 2.8 m, the long-term data transmission and power supply can be realized by using cables, and the foldable probe design makes the system deployment more flexible.

## 7. Conclusions

In this study, based on the sensing technology combining electricity and mechanics, we developed an underwater adaptive penetration system (UAPS/ILMM) capable of multi-parameter monitoring for the needs of marine science and ocean engineering. It is mainly composed of a platform carrier, a foldable multi-sensor probe, and an underwater adaptive penetration device. This design overcomes the technical challenges of long-stroke probe penetration and simultaneous multi-parameter data acquisition under the premise of a relatively short total height of 2.8 m and a relatively light total weight of 1450 kg. UAPS/ILMM can achieve a penetration depth of 5 m below the surface of the seabed, and by increasing and decreasing the rods according to the actual situation, it can also simultaneously obtain data such as seabed elevation, sediment pore water pressure, and excess pore water pressure. Static analysis of the frame and seal chamber shows that the frame is evenly stressed and within the yield strength of the material. Laboratory tests on the self-potential and pore water pressure sensors confirmed the feasibility of the sensors to monitor the sediment-water-air interface and the stability of monitoring pore water pressure. Finally, we tested the UAPS/ILMM in a harbor environment. Preliminary results demonstrated the system’s operability and effectiveness. The underwater automatic splicing equipment of rods and the multi-parameter integrated monitoring probe proposed in this paper can offer new insights for the development and application of in situ seabed monitoring equipment. However, it still does not realize the synchronous monitoring of the seawater environment and the operation that requires the deployment of ribbon cables to achieve penetration. There is still a lot of room for improvement in multi-sensor integration and underwater wireless signal transmission in the future.

## Figures and Tables

**Table 1:** Technical indexes of UAPS/ILMM.

Item	Parameter and Technical Index
Size	2800 × 1800 × 2800 mm
Weight	1450 kg (without ballast)
Usage environment	−5~40 °C
Working water depth	0~500 m
Body material	Stainless steel (316 L)
Penetration method	Friction penetration
Penetration depth	0~5 m (according to site conditions)
Sensors	Self-potential sensor (0–2000 mV range, 0.01 mV resolution); Pore water pressure sensor (0–10 MPa range, 0.1% F.S. accuracy)
Monitorable parameters	Seabed elevation; Pore water pressure; Excess pore water pressure

**Table 2:** Comparison between the proposed UAPS/ILMM and other systems.

System	Data Transmission Method	Power Transmission Method	Monitoring Objects	Structural Features	Penetration Depth	Long-Term Real-Time Monitoring	Deployment Flexibility
UAPS/ILMM	Real-time/ Self-contained	Cable transmission in real time	Seabed; Sediments	1450 kg weight; 2.8 m high	5.0 m (Can be increased or decreased)	Yes	High
SEEGeo [33,34]	Real-time/ Self-contained	Cable transmission in real time	Sediments	2800 kg weight; 2.2 m high	3.5 m	Yes	High
Seabed Monitoring System [35]	Self-contained	Self- contained	Seabed; Seawater	800 kg weight; 2.2 m high	0 m	Based on self-contained battery capacity	High
In Situ Test System [26,27,28,29,36]	Real-time/ Self-contained	Cable transmission in real time	Sediments	3500–5500 kg weight; 2.4–3.5 m high	0–50.0 m	No	Medium
Sediment Pore Water Pressure Monitoring System [20,21,22,23,24,25]	Self-contained	Self- contained	Sediments	7.7–500 kg weight; 3.0–8.0 m high	0–4.8 m	Based on self-contained battery capacity	Medium

## Data Availability

All data during this study appear in this submitted article.
